# Green-synthesized silver nanoparticle hydrogels for biofilm-infected wounds: Bridging sustainability and clinical translation

**DOI:** 10.3389/fphar.2025.1694144

**Published:** 2025-10-24

**Authors:** M. Rifqi Fadillah Muslim, Lutfi Chabib, Betty Ekawati Suryaningsih, Farida Hayati, Viviane Annisa

**Affiliations:** ^1^ Department of Pharmacy, Faculty of Mathematics and Natural Sciences, Islamic University of Indonesia, Yogyakarta, Indonesia; ^2^ Department of Medicine, Faculty of Medicine, Islamic University of Indonesia, Yogyakarta, Indonesia

**Keywords:** green-synthesized, silver nanoparticle, hydrogels, biofilm-infected wound, translation

## 1 Introduction

Chronic wound infections remain a serious global public health concern, contributing to frequent hospitalizations, delayed healing, and increased mortality. A key barrier to recovery is the presence of biofilms. Biofilms are structured microbial communities embedded within an extracellular polymeric substance (EPS) matrix that restricts antibiotic diffusion and penetration, diminishing antibacterial efficacy (Zhao et al., 2022). Conventional dressings such as gauze and bandages often fail to maintain the moist environment required for optimal tissue repair and can adhere to the wound bed, causing pain and additional tissue trauma during removal (Kim et al., 2024). In contrast, contemporary practice increasingly favors moist wound healing, for which hydrogels serve as suitable matrices to regulate exudate, preserve moisture, and facilitate the incorporation and delivery of antimicrobial agents. Recent meta-analytic evidence also suggests that hydrogel-based dressings can outperform traditional dressings for chronic diabetic foot ulcers (Zhao et al., 2024). Beyond moisture management, immunomodulation, and microenvironment engineering, such as hyaluronic acid–based hydrogels, are gaining relevance for promoting angiogenesis in non-healing wounds (Liu et al., 2020).

Silver nanoparticles (AgNPs) have attracted considerable interest in this landscape due to their multi-target antibacterial properties and anti-biofilm potential. Therefore, embedding AgNPs within hydrogel matrices is a promising next-generation dressing for biofilm-infected wounds (Khalifa et al., 2025). While green synthesis approaches offer a more sustainable route to AgNPs production, they face practical hurdles, particularly batch-to-batch reproducibility and scalability before clinical adoption. Accordingly, successful translation will require standardized manufacturing and characterization protocols, alongside rigorously controlled clinical evaluations.

## 2 Discussion

### 2.1 The promise of green-synthesized AgNP hydrogel

**FIGURE 1:**
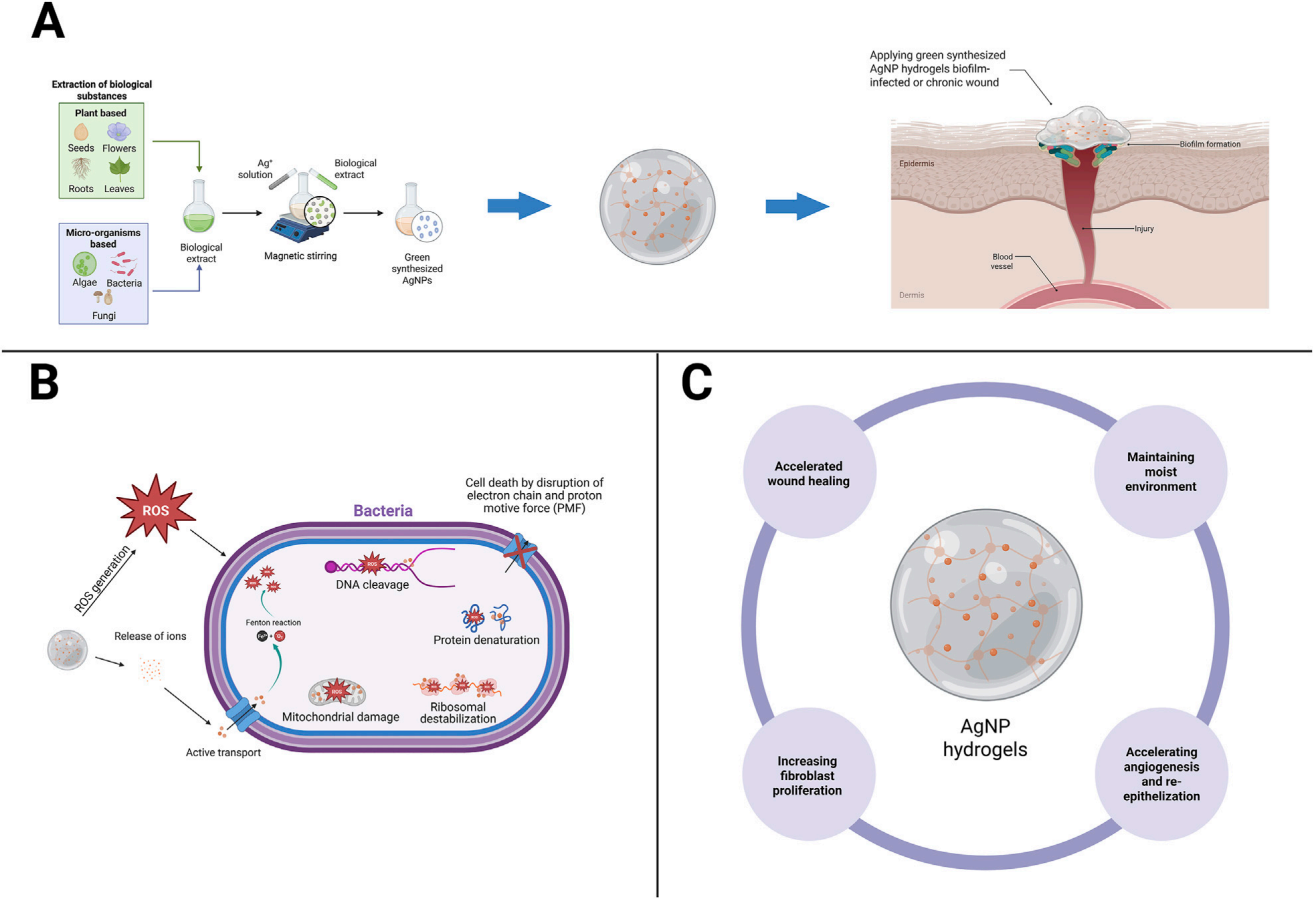
Mechanistic pathways of green-synthesized AgNP hydrogels in biofilm-infected wound healing. **(A)** Green synthesis of AgNPs using biological sources (plants, algae, bacteria, fungi) yields nanoparticles that can be incorporated into hydrogels. **(B)** The AgNP hydrogel releases silver ions (Ag^+^) in a controlled manner, penetrating the extracellular polymeric matrix of biofilms, generating reactive oxygen species (ROS), collectively leading to bacterial death and biofilm inhibition. **(C)** The AgNP hydrogel accelerate wound closure by maintaining moisture balance, stimulating fibroblast proliferation, enhancing angiogenesis, and promoting re-epithelialization. *Created with BioRender.com (licensed to the corresponding author’s institution)*.

Hydrogels incorporating AgNPs address three key gaps in managing biofilm-infected chronic wounds: maintaining a conducive moist environment, overcoming antibiotic diffusion barriers within the biofilm matrix, and providing multi-target antimicrobial activity at the site of infection. Clinically, a recent meta-analysis demonstrated that hydrogels are more effective than conventional dressings for diabetic foot ulcers (DFUs), strengthening the rationale for using hydrogels as antimicrobial carrier matrices in chronic wounds (Zhao et al., 2024). Mechanistically, AgNPs exhibit various activities, such as disrupting membranes, inactivating enzymes/proteins through interactions with thiol groups, disrupting DNA, and inducing oxidative stress, which is detrimental to bacteria and biofilms, leading to a marked increase in antibiotic tolerance. Strong *in vivo* evidence demonstrates that AgNP hydrogels are capable of eradicating mature biofilms and accelerating wound repair compared to controls, with faster wound closure and re-epithelialization in a *Staphylococcus aureus* infection model (Haidari et al., 2021a). The mechanism of green-synthesized AgNP hydrogels in biofilm-infected wound healing can be seen in Figure 1.

The advantages of green synthesis using plant metabolites as reducers/stabilizers include the potential for biogenic capping and a more sustainable process pathway, while still producing significant biological effects when green-synthesized AgNPs are integrated into polymer hydrogels. In several *in vivo* formulations over 14 days, the green-synthesized AgNP hydrogel composite was biocompatible and accelerated wound healing (Shehzad et al., 2025). On the other hand, precise silver ions (Ag^+^) dose/release adjustments are required to minimize ineffective subtherapeutic levels, while excessive release risks cytotoxicity and inhibits epithelialization. Therefore, formulation development requires a standardized testing framework, such as ASTM E2799 for minimum biofilm eradication concentration (MBEC), to assess *Pseudomonas aeruginosa* biofilm eradication in a standardized and comparable manner across laboratories. Finally, given the antimicrobial-tolerant nature of biofilms, the AgNP hydrogel combination approach demonstrated in in vitro/*in vivo* studies provides a strong rationale for biofilm-inhibitory combinations over single therapies as a pathway that aligns with the clinical needs of chronic wounds infected with resistant pathogens.

### 2.2 Beyond sustainability: green and chemical synthesis

The central question is not “green or chemical,” but how to make a product at consistent quality and scale. On the chemical side, continuous-flow and microreactor platforms offer tighter control over particle size and size distribution, low batch-to-batch variability, and the option for in-line monitoring, which is critical for reproducibility and scale-up. Studies using continuous-flow reactors show that channel geometry and operating conditions can be tuned to precisely AgNPs size, similar control has been demonstrated in microwave-assisted continuous processes and modern microfluidic systems. In practical terms, these engineering advantages map directly onto Good Manufacturing Practice (GMP) expectations and a Quality-by-Design (QbD) mindset, where quality is built into the process rather than inspected only at the end (Ghosh et al., 2024). By contrast, green synthesis which uses plant or microbial metabolites as reducing and capping agents offers a more sustainable processing route and can yield biogenic surface chemistries that are friendly to living tissue. Importantly, this is more than a theoretical benefit, *in vivo* wound models have reported faster wound closure, improved re-epithelialization, and better infection control when green-synthesized AgNPs are delivered in gels or hydrogels (including diabetic and burn models) (Cheng et al., 2024; Gaikwad et al., 2022; Sari et al., 2025). At the same time, head-to-head experiments indicate that the biological source and the reduction pathway materially influence bioactivity and toxicity. For example, AgNPs made from clove extract can behave differently from sodium borohydride (NaBH_4_)-reduced or glutathione (GSH)-capped particles. The takeaway is clear, green synthesis can be biologically effective, but its profile is highly sensitive to phytochemical composition, a major driver of batch variability that must be controlled before moving to industrial manufacturing.

Safety and residuals need a balanced view. With chemical reduction (e.g., NaBH_4_), particles are often more uniform and easier to reproduce. However, if not adequately removed, residual reagents or impurities may increase cytotoxicity, underscoring the importance of purification for medical use. Conversely, green-synthesized AgNPs are not automatically non-toxic. Their safety is dose-dependent and shaped by size, shape, and silver ions (Ag^+^) release. In practice, both routes demand rigorous process control and characterization, including particle size and distribution, surface charge (zeta potential), morphology, Ag^+^ loading and release kinetics, plus standardized *in vitro* cytotoxicity testing before animal or human evaluation (Gil-Korilis et al., 2023; Laib et al., 2025).

Given these realities, a hybrid pathway is compelling: (i) standardize green precursors by defining quantitative phytochemical markers; (ii) run biosynthetic feeds on continuous-flow platforms to suppress variability and lock in size; and (iii) embed Process Analytical Technology (PAT) and Design of Experiments (DoE) within a QbD framework to tie process parameters directly to critical quality attributes (CQAs). This strategy rationally combines sustainability (green) with manufacturing discipline (chemical/process engineering), producing a regulator-ready data trail and accelerating translation toward targeted clinical trials. The enabling technologies for continuous flow/microreactor manufacturing already exist (Ghosh et al., 2024). The next step is to apply them to green systems so that reproducibility approaches chemical routes without sacrificing the sustainability benefits that motivate green synthesis in the first place.

### 2.3 Bridging safety, regulation, and clinical translation

Despite promising preclinical findings, the clinical development of AgNP hydrogels is still constrained by unresolved efficacy, safety, and regulatory acceptance issues. A recent meta-analysis on diabetic foot ulcers (DFUs) reported that silver dressings improved healing rates and shortened healing times compared with controls (Xie et al., 2025). However, substantial heterogeneity across trials indicates the need for more rigorous, head-to-head comparisons with current best practices. Similarly, the SilvrSTAT gel randomized controlled trial (RCT) showed faster healing than conventional dressings (Essa et al., 2023). However, the limitations of comparator choice and sample size highlight the necessity of larger RCTs across diverse chronic wounds complicated by biofilm.

Conventional silver and zinc-oxide dressings provide transient antibacterial activity. They are effective against planktonic bacteria but show limited penetration into mature biofilms due to rapid ion depletion and surface confinement. In contrast, AgNP hydrogels enable sustained Ag^+^ release and diffusion through the biofilm matrix, facilitating more profound disruption of bacterial communities. While direct head-to-head clinical data remain limited, comparative preclinical results suggest that AgNP-based systems maintain antimicrobial efficacy for longer durations and at lower silver concentrations than commercial Ag^+^ plasters. For example, an AgNP hydrogel (200 μg/g) achieved 46% wound closure compared to 20% in the silver sulfadiazine control in *S. aureus* biofilm model (Haidari et al., 2021a), and injectable AgNP hydrogels have demonstrated sustained bactericidal activity with minimal cytotoxicity (Zhao et al., 2025). Thus, the rationale for AgNP hydrogel development lies not in replacing existing silver dressings but in overcoming their kinetic and diffusion limitations within biofilm-infected wounds.

Safety should not be assumed but demonstrated. *In vitro* and *ex vivo* studies of AgNP hydrogel formulations have shown that skin cell viability is preserved within controlled exposure ranges, whereas excessive exposure leads to cytotoxicity and impaired cell migration, underscoring the importance of regulating Ag^+^ release (Cunha et al., 2024). *In vivo* human studies further indicate that Ag^+^ can penetrate the dermis but are not systemically detected. This supports the feasibility of safe topical use if drug release and dosage are carefully managed (George et al., 2014).

From a regulatory standpoint, AgNP hydrogels, particularly those produced by green synthesis require robust process and product standards. This includes defining raw material markers such as phytochemical profiles, implementing process controls, and establishing specifications for particle size, zeta potential, and Ag^+^ release kinetics. Clinical and *in vivo* data show that Ag^+^ can accumulate in the dermis and even raise serum levels in patients with extensive burns, reinforcing the need for limits on exposure duration and systematic monitoring (Nešporová et al., 2020). Anti-biofilm claims must also be validated using standardized methods, such as minimum biofilm eradication concentration (MBEC) testing, to ensure cross-study comparability. Reflecting these concerns, the FDA’s 2022 guidance on nanomaterial-containing products emphasized a risk-based approach. Product quality must be built into the design through rigorous characterization, linkage of process parameters to critical quality attributes, and documented control of active ingredient release. Ultimately, translation demands alignment between preclinical rigor, regulatory standards, and clinical trial design. The next step is not more proof-of-concept studies, but head-to-head RCTs in biofilm-infected wounds with endpoints that matter to patients: closure time, recurrence of infection, pain, and quality of life. Only through this integration can AgNP hydrogels shift from experimental promise to clinical practice.

### 2.4 Outlook and opportunities ahead

Going forward, the future of AgNP hydrogels will depend on the ability to translate consistent laboratory performance into reliable clinical outcomes. Several *in vivo* experiments have shown promising results. (Haidari et al., 2021b). demonstrated that a temperature-responsive AgNP hydrogel accelerated wound healing in infected mice while reducing inflammation compared to silver sulfadiazine. These results suggest that antimicrobial potential and biocompatibility can be harnessed simultaneously when Ag^+^ kinetics are intelligently regulated.

However, the clinical pathway remains narrow. Consistency across batches, validated Ag^+^ release kinetics, and transparent manufacturing protocols will determine whether these formulations can move beyond research. Regulators will demand proof of reproducibility, not just efficacy, and long-term studies are needed to understand systemic Ag^+^ exposure. Future clinical trials should be designed with patient-centered endpoints such as healing time, infection recurrence, and comfort, rather than simply wound size. Understanding these factors will require multidisciplinary collaboration between materials scientists, pharmacologists, and regulatory agencies, ideally beginning in the preclinical phase, rather than later.

Clinically, the potential of AgNP hydrogels extends far beyond chronic wound management. Their ability to deliver local antimicrobial action within a moist, biocompatible matrix makes them suitable for post-surgical dressings, diabetic ulcers, and burns, where biofilm recurrence often complicates healing. The controlled release of Ag^+^ can reduce the need for systemic antibiotics and lower the risk of resistance development, aligning with global antimicrobial stewardship initiatives. Early clinical experience has also reported improved pain control, odor reduction, and exudate management, which improve patient comfort and compliance.

In the near future, combining AgNP hydrogels with growth factors, anti-inflammatory agents, or regenerative peptides could create a multifunctional framework capable of combating infection and promoting tissue repair. Their malleable structure also supports customized wound care, adapting to anatomical features and exudate profiles. The challenge lies in achieving a delicate balance between antimicrobial efficacy and long-term safety. However, with rigorous standardization, validated release control, and well-designed clinical evaluations, AgNP hydrogels have the potential to progress from an experimental adjunctive therapy to a first-line clinical therapy for biofilm-infected and chronic wounds.

## 3 Conclusion

Green-synthesized AgNP hydrogels stand at a promising crossroads between sustainable chemistry and clinical innovation to address biofilm-infected chronic wounds. Preclinical findings already demonstrate translational potential such as achieving moisture balance, disrupting biofilms, and maintaining tissue compatibility within a single therapeutic platform. However, the progress will not be advanced by further proof-of-concept studies alone, but by disciplined reproducibility and rigorously designed clinical trials. Meaningful advancement requires standardized phytochemical markers for biosynthetic precursors, stringent process control over particle characteristics and Ag^+^ release kinetics, and cross-laboratory anti-biofilm assays that enable genuine comparability rather than narrative claims.

From both pharmacological and regulatory perspectives, adopting a Quality-by-Design framework is now essential. Process parameters must be explicitly linked to critical quality attributes, and dose–release profiles verified for both efficacy and safety. Biofilm eradication metrics should also reflect patient-centered outcomes such as healing time, recurrence, pain, and overall quality of life. This approach will help the field move beyond inconsistent evidence and allow AgNP hydrogels to be fairly compared with existing modern moist dressings. When sustainability and manufacturing rigor are pursued together rather than separately, green-synthesized AgNP hydrogels may realistically evolve from promising laboratory concepts into clinically reliable, regulator-ready therapies for biofilm-infected chronic wounds.
